# Dopaminium perchlorate

**DOI:** 10.1107/S1600536808035666

**Published:** 2008-11-08

**Authors:** Davar M. Boghaei, Sahar Baniyaghoob, Mohammad Mahdi Najafpour, Vickie McKee

**Affiliations:** a Department of Chemistry, Sharif University of Technology, PO Box 11155-8639, Tehran, Iran; b Department of Chemistry, Loughborough University, Leicestershire LE11 3TU, England

## Abstract

In the title compound [systematic name: 2-(3,4-dihydroxy­phen­yl)ethanaminium perchlorate], C_8_H_12_NO_2_^+^·ClO_4_^−^, the cations and anions are linked into three-dimensional structure *via* inter­molecular N—H⋯O and O—H⋯O hydrogen bonds.

## Related literature

For related crystal structures, see: Bergin & Carlström (1968 ▶); Giesecke (1980 ▶). For details of the pharmacological properties of dopamine, see Salamone & Correa (2002 ▶).
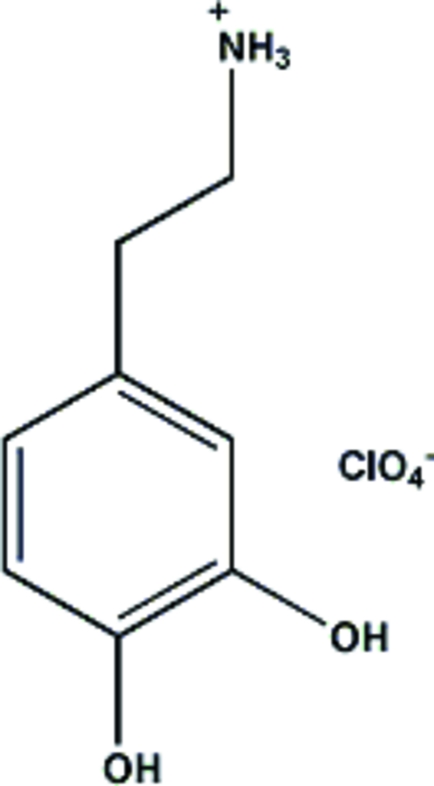


## Experimental

### 

#### Crystal data

C_8_H_12_NO_2_^+^·ClO_4_^−^
*M* *_r_* = 253.64
Triclinic, 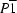

*a* = 7.4925 (3) Å
*b* = 8.2254 (3) Å
*c* = 8.9524 (4) Å
α = 106.910 (1)°
β = 94.186 (1)°
γ = 101.206 (1)°
*V* = 512.85 (4) Å^3^
*Z* = 2
Mo *K*α radiation
μ = 0.39 mm^−1^
*T* = 150 (2) K
0.30 × 0.15 × 0.06 mm

#### Data collection

Bruker APEXII CCD diffractometer
Absorption correction: multi-scan (*SADABS*; Sheldrick, 2003 ▶) *T*_min_ = 0.893, *T*_max_ = 0.977
6199 measured reflections
3146 independent reflections
2858 reflections with *I* > 2σ(*I*)
*R*_int_ = 0.018

#### Refinement

*R*[*F* ^2^ > 2σ(*F* ^2^)] = 0.036
*wR*(*F* ^2^) = 0.105
*S* = 1.08
3146 reflections
145 parameters
H-atom parameters constrained
Δρ_max_ = 0.49 e Å^−3^
Δρ_min_ = −0.45 e Å^−3^

### 

Data collection: *APEX2* (Bruker, 2005 ▶); cell refinement: *SAINT* (Bruker, 2005 ▶); data reduction: *SAINT*; program(s) used to solve structure: *SHELXS97* (Sheldrick, 2008 ▶); program(s) used to refine structure: *SHELXL97* (Sheldrick, 2008 ▶); molecular graphics: *SHELXTL* (Sheldrick, 2008 ▶); software used to prepare material for publication: *SHELXTL*.

## Supplementary Material

Crystal structure: contains datablocks I, global. DOI: 10.1107/S1600536808035666/cv2470sup1.cif

Structure factors: contains datablocks I. DOI: 10.1107/S1600536808035666/cv2470Isup2.hkl

Additional supplementary materials:  crystallographic information; 3D view; checkCIF report

## Figures and Tables

**Table 1:** Hydrogen-bond geometry (Å, °)

*D*—H⋯*A*	*D*—H	H⋯*A*	*D*⋯*A*	*D*—H⋯*A*
O1—H1*O*⋯O11	0.85	2.16	2.9065 (14)	146
O2—H2*O*⋯O11^i^	0.85	1.96	2.7936 (15)	164
N1—H1*A*⋯O1^ii^	0.91	2.07	2.8822 (14)	148
N1—H1*B*⋯O14^iii^	0.91	1.93	2.8317 (16)	169
N1—H1*C*⋯O12^iv^	0.91	2.11	2.8002 (16)	132
N1—H1*C*⋯O2^iv^	0.91	2.39	3.0512 (16)	130

Symmetry codes: (i) 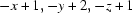; (ii) 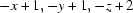; (iii) 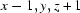; (iv) 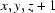.

**Fig. 1.**
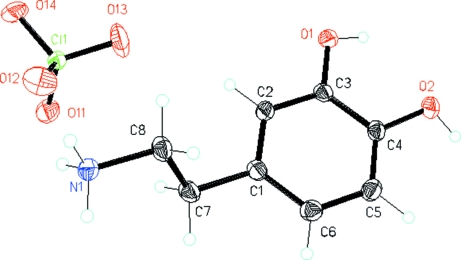
The molecular structure of (I) showing the atomic labels and displacement ellipsoids for non-H atoms drawn at the 50% probability level.

## supplementary crystallographic information

### Comment

Many neuro transmitters have been discovered over the past century, such as serotonin, norepinephrine, substance P and dopamine. Dopamine is synthesized in the cenral brain from tyrosine. Dopamine has been considered as an important signal transmitter between the neurons and muscles (Salamone & Correa, 2002). Herewith we present the crystal structure of the title compound (I).

In (I) (Fig. 1), all bond lengths and angles are normal (Giesecke, 1980, Bergin & Carlström, 1968). The torsion angles C6—C1—C7—C8 and C1—C7—C8—N1 are 111.9 (1)° and 179.9 (6)°, respectively, showing that C1—C7—C8—N1 chain is almost fully extended, forming a plane that is nearly orthogonal to the plane of the ring. The crystal packing is stabilized by an extensive network of O—H···O and N—H···O hydrogen bonds (Table 1).

### Experimental

The title compound was prepared by dissolving dopamine hydrochloride (2 mmol, 379 mg) and NaClO_4_.H_2_O (2 mmol, 280 mg) in water/HClO_4_ (1mM, 10 ml). The mixture was stirred for about 2 h at room temperature. This solution yielded colourless crystals of (I) after 10 d.

### Refinement

All H atoms atoms were placed in calculated positions and refined using the riding model approximation, with C—H = 0.95–1.0 Å, O—H = 0.85 Å, N—H = 0.91 Å, and with *U*_iso_(H) = 1.2Ueq(C) or 1.5Ueq(N). The isotropic displacement parameter of the hydroxy H atoms were fixed to 0.04 Å^2 ^

### Crystal data

**Table:**

C_8_H_12_NO_2_^+^·ClO_4_^–^	*Z* = 2
*M**_r_* = 253.64	*F*_000_ = 264
Triclinic, *P*1	*D*_x_ = 1.642 Mg m^−^^3^
Hall symbol: -P1	Mo *K*α radiation λ = 0.71073 Å
*a* = 7.4925 (3) Å	Cell parameters from 3633 reflections
*b* = 8.2254 (3) Å	θ = 2.4–31.6º
*c* = 8.9524 (4) Å	µ = 0.39 mm^−^^1^
α = 106.910 (1)º	*T* = 150 (2) K
β = 94.186 (1)º	Plate, colourless
γ = 101.206 (1)º	0.30 × 0.15 × 0.06 mm
*V* = 512.85 (4) Å^3^	

### Data collection

**Table:**

Bruker APEXII CCD diffractometer	3146 independent reflections
Radiation source: fine-focus sealed tube	2858 reflections with *I* > 2σ(*I*)
Monochromator: graphite	*R*_int_ = 0.018
*T* = 150(2) K	θ_max_ = 31.7º
φ and ω scans	θ_min_ = 2.4º
Absorption correction: multi-scan(SADABS; Sheldrick, 2003)	*h* = −10→10
*T*_min_ = 0.893, *T*_max_ = 0.977	*k* = −12→11
6199 measured reflections	*l* = −12→12

### Refinement

**Table:**

Refinement on *F*^2^	Secondary atom site location: difference Fourier map
Least-squares matrix: full	Hydrogen site location: inferred from neighbouring sites
*R*[*F*^2^ > 2σ(*F*^2^)] = 0.036	H-atom parameters constrained
*wR*(*F*^2^) = 0.105	*w* = 1/[σ^2^(*F*_o_^2^) + (0.0559*P*)^2^ + 0.2163*P*] where *P* = (*F*_o_^2^ + 2*F*_c_^2^)/3
*S* = 1.09	(Δ/σ)_max_ < 0.001
3146 reflections	Δρ_max_ = 0.49 e Å^−^^3^
145 parameters	Δρ_min_ = −0.45 e Å^−^^3^
Primary atom site location: structure-invariant direct methods	Extinction correction: none

### Special details

**Table:**

Geometry. All e.s.d.'s (except the e.s.d. in the dihedral angle between two l.s. planes) are estimated using the full covariance matrix. The cell e.s.d.'s are taken into account individually in the estimation of e.s.d.'s in distances, angles and torsion angles; correlations between e.s.d.'s in cell parameters are only used when they are defined by crystal symmetry. An approximate (isotropic) treatment of cell e.s.d.'s is used for estimating e.s.d.'s involving l.s. planes.
Refinement. Refinement of *F*^2^ against all reflections. The weighted *R*-factor *wR* andgoodness of fit *S* are based on *F*^2^, conventional *R*-factors *R* are basedon *F*, with *F* set to zero for negative *F*^2^. The threshold expression of*F*^2^ > σ(*F*^2^) is used only for calculating *R*-factors(gt) *etc*. and isnot relevant to the choice of reflections for refinement. *R*-factors basedon *F*^2^ are statistically about twice as large as those based on *F*, and *R*-factors based on all data will be even larger.

### Fractional atomic coordinates and isotropic or equivalent isotropic displacement parameters (Å^2^)

**Table:**

	*x*	*y*	*z*	*U*_iso_*/*U*_eq_	
Cl1	0.78299 (4)	0.71277 (4)	0.38696 (3)	0.01882 (9)	
O11	0.78911 (17)	0.88541 (14)	0.49463 (13)	0.0299 (2)	
O12	0.61768 (16)	0.59722 (16)	0.39753 (16)	0.0370 (3)	
O13	0.7897 (2)	0.7193 (2)	0.23036 (14)	0.0436 (3)	
O14	0.93581 (16)	0.65238 (18)	0.43983 (14)	0.0373 (3)	
C1	0.28726 (18)	0.78530 (16)	1.02178 (15)	0.0185 (2)	
C2	0.45074 (18)	0.77363 (16)	0.95805 (14)	0.0185 (2)	
H2	0.5479	0.7464	1.0135	0.022*	
C3	0.47193 (17)	0.80155 (16)	0.81453 (14)	0.0177 (2)	
O1	0.63545 (13)	0.79282 (13)	0.75554 (11)	0.02136 (19)	
C4	0.32907 (18)	0.84083 (16)	0.73205 (14)	0.0188 (2)	
O2	0.36279 (15)	0.86353 (13)	0.58877 (11)	0.0240 (2)	
C5	0.16751 (18)	0.85522 (17)	0.79484 (16)	0.0212 (2)	
H5	0.0713	0.8842	0.7397	0.025*	
C6	0.14636 (18)	0.82696 (17)	0.93990 (16)	0.0208 (2)	
H6	0.0352	0.8362	0.9829	0.025*	
C7	0.2661 (2)	0.75345 (17)	1.17802 (15)	0.0215 (2)	
H7A	0.1522	0.7850	1.2147	0.026*	
H7B	0.3713	0.8279	1.2575	0.026*	
C8	0.2576 (2)	0.56300 (17)	1.16162 (15)	0.0222 (3)	
H8A	0.1526	0.4892	1.0815	0.027*	
H8B	0.3714	0.5320	1.1244	0.027*	
N1	0.23650 (15)	0.52615 (15)	1.31419 (13)	0.0200 (2)	
H1A	0.2317	0.4112	1.2999	0.030*	
H1B	0.1310	0.5528	1.3479	0.030*	
H1C	0.3340	0.5921	1.3875	0.030*	
H2O	0.2968	0.9273	0.5626	0.040*	
H1O	0.6356	0.8281	0.6748	0.040*	

### Atomic displacement parameters (Å^2^)

**Table:**

	*U*^11^	*U*^22^	*U*^33^	*U*^12^	*U*^13^	*U*^23^
Cl1	0.01669 (14)	0.02486 (16)	0.01828 (15)	0.00806 (11)	0.00411 (10)	0.00922 (11)
O11	0.0394 (6)	0.0222 (5)	0.0305 (5)	0.0104 (4)	0.0048 (4)	0.0096 (4)
O12	0.0253 (5)	0.0348 (6)	0.0453 (7)	−0.0014 (5)	0.0097 (5)	0.0083 (5)
O13	0.0561 (8)	0.0597 (8)	0.0200 (5)	0.0123 (7)	0.0065 (5)	0.0201 (5)
O14	0.0303 (6)	0.0537 (7)	0.0342 (6)	0.0287 (5)	0.0029 (4)	0.0113 (5)
C1	0.0249 (6)	0.0149 (5)	0.0170 (5)	0.0059 (4)	0.0049 (4)	0.0058 (4)
C2	0.0241 (6)	0.0173 (5)	0.0166 (5)	0.0079 (4)	0.0028 (4)	0.0070 (4)
C3	0.0224 (5)	0.0153 (5)	0.0168 (5)	0.0066 (4)	0.0046 (4)	0.0052 (4)
O1	0.0248 (5)	0.0250 (5)	0.0206 (4)	0.0118 (4)	0.0087 (3)	0.0114 (4)
C4	0.0256 (6)	0.0168 (5)	0.0150 (5)	0.0061 (4)	0.0016 (4)	0.0059 (4)
O2	0.0327 (5)	0.0276 (5)	0.0174 (4)	0.0132 (4)	0.0047 (4)	0.0116 (4)
C5	0.0229 (6)	0.0209 (5)	0.0213 (6)	0.0070 (5)	0.0005 (4)	0.0079 (5)
C6	0.0215 (6)	0.0199 (5)	0.0229 (6)	0.0063 (4)	0.0052 (5)	0.0079 (5)
C7	0.0301 (6)	0.0193 (5)	0.0194 (6)	0.0091 (5)	0.0094 (5)	0.0085 (4)
C8	0.0331 (7)	0.0189 (5)	0.0168 (5)	0.0077 (5)	0.0048 (5)	0.0077 (4)
N1	0.0215 (5)	0.0229 (5)	0.0208 (5)	0.0085 (4)	0.0064 (4)	0.0118 (4)

### Geometric parameters (Å, °)

**Table:**

Cl1—O13	1.4225 (11)	O2—H2O	0.8540
Cl1—O12	1.4347 (11)	C5—C6	1.3989 (18)
Cl1—O14	1.4355 (11)	C5—H5	0.9500
Cl1—O11	1.4559 (11)	C6—H6	0.9500
C1—C6	1.3939 (18)	C7—C8	1.5185 (18)
C1—C2	1.3968 (18)	C7—H7A	0.9900
C1—C7	1.5099 (17)	C7—H7B	0.9900
C2—C3	1.3842 (16)	C8—N1	1.4954 (16)
C2—H2	0.9500	C8—H8A	0.9900
C3—O1	1.3753 (15)	C8—H8B	0.9900
C3—C4	1.3975 (18)	N1—H1A	0.9100
O1—H1O	0.8537	N1—H1B	0.9100
C4—C5	1.3826 (19)	N1—H1C	0.9100
C4—O2	1.3828 (15)		
			
O13—Cl1—O12	111.25 (8)	C6—C5—H5	120.2
O13—Cl1—O14	111.08 (8)	C1—C6—C5	120.47 (12)
O12—Cl1—O14	107.81 (8)	C1—C6—H6	119.8
O13—Cl1—O11	110.40 (8)	C5—C6—H6	119.8
O12—Cl1—O11	108.15 (7)	C1—C7—C8	110.27 (10)
O14—Cl1—O11	108.02 (7)	C1—C7—H7A	109.6
C6—C1—C2	119.24 (11)	C8—C7—H7A	109.6
C6—C1—C7	121.09 (11)	C1—C7—H7B	109.6
C2—C1—C7	119.67 (11)	C8—C7—H7B	109.6
C3—C2—C1	120.37 (12)	H7A—C7—H7B	108.1
C3—C2—H2	119.8	N1—C8—C7	111.89 (10)
C1—C2—H2	119.8	N1—C8—H8A	109.2
O1—C3—C2	119.37 (11)	C7—C8—H8A	109.2
O1—C3—C4	120.57 (11)	N1—C8—H8B	109.2
C2—C3—C4	120.06 (11)	C7—C8—H8B	109.2
C3—O1—H1O	109.3	H8A—C8—H8B	107.9
C5—C4—O2	124.27 (11)	C8—N1—H1A	109.5
C5—C4—C3	120.16 (11)	C8—N1—H1B	109.5
O2—C4—C3	115.56 (11)	H1A—N1—H1B	109.5
C4—O2—H2O	111.4	C8—N1—H1C	109.5
C4—C5—C6	119.69 (12)	H1A—N1—H1C	109.5
C4—C5—H5	120.2	H1B—N1—H1C	109.5

### Hydrogen-bond geometry (Å, °)

**Table:**

*D*—H···*A*	*D*—H	H···*A*	*D*···*A*	*D*—H···*A*
O1—H1O···O11	0.85	2.16	2.9065 (14)	146
O2—H2O···O11^i^	0.85	1.96	2.7936 (15)	164
N1—H1A···O1^ii^	0.91	2.07	2.8822 (14)	148
N1—H1B···O14^iii^	0.91	1.93	2.8317 (16)	169
N1—H1C···O12^iv^	0.91	2.11	2.8002 (16)	132
N1—H1C···O2^iv^	0.91	2.39	3.0512 (16)	130

Symmetry codes: (i) −*x*+1, −*y*+2, −*z*+1; (ii) −*x*+1, −*y*+1, −*z*+2; (iii) *x*−1, *y*, *z*+1; (iv) *x*, *y*, *z*+1.
